# Community ‐and hospital laboratory‐based surveillance for respiratory viruses

**DOI:** 10.1111/irv.12387

**Published:** 2016-03-27

**Authors:** Philip Zachariah, Susan Whittier, Carrie Reed, Philip LaRussa, Elaine L. Larson, Celibell Y. Vargas, Lisa Saiman, Melissa S. Stockwell

**Affiliations:** ^1^ Columbia University Medical CenterNew YorkNYUSA; ^2^ New York‐Presbyterian HospitalNew YorkNYUSA; ^3^ Centers for Disease Control and PreventionAtlantaGAUSA

**Keywords:** Influenza, surveillance, viruses

## Abstract

Traditional surveillance for respiratory viruses relies on symptom detection and laboratory detection during medically attended encounters for acute respiratory infection/influenza‐like illness (ARI/ILI). Ecological momentary reporting using text messages is a novel method for surveillance. This study compares respiratory viral activity detected through longitudinal community‐based surveillance using text message responses for sample acquisition and testing to respiratory viral activity obtained from hospital laboratory data from the same community. We demonstrate a significant correlation between community‐ and hospital laboratory‐based surveillance for most respiratory viruses, although the relative proportions of viruses detected in the community and hospital differed significantly.

## Background

Traditionally, surveillance for respiratory viruses in the U.S.A. relies on symptom detection on laboratory testing during medically attended encounters or hospitalizations for acute respiratory infection/influenza‐like illness (ARI/ILI).^1^ These methods may not represent population‐level viral activity, as they rely on individuals seeking medical care, because of either personal health behaviors or severity of illness. Surveillance of ARI/ILI in community‐based settings may better represent population‐based respiratory viral activity. Direct comparison of viral activity in the community versus in hospitalized patients (derived from the same community) could further help determine the impact of specific viruses and identify the utility of including community‐based samples for ARI/ILI surveillance.

Text messaging is a method of ecological momentary reporting that can be used for population‐based infectious disease surveillance that has been pilot‐tested once in Mexico for pandemic influenza and for provider‐reported ILI incidence data in Madagascar.^2^, ^3^ We recently demonstrated the feasibility of using text messaging for community‐based surveillance of ARI/ILI.^4^ In the current study, we compare respiratory viral activity detected through longitudinal community‐based surveillance using text message responses for targeted sample acquisition and diagnostic testing to respiratory viral activity obtained from hospital laboratory‐based data (from the same community). We hypothesized that viral activity obtained from community‐based surveillance would correspond to that obtained from laboratory‐based surveillance and demonstrate similar viral strain distributions.

## Methods

### Community‐based surveillance using text messaging

Community‐based surveillance for respiratory viruses was conducted from January 2013 to June 2014 in Washington Heights/Inwood, a diverse low‐income neighborhood served by Columbia University Medical Center (CUMC) in New York City (NYC), as part of an ongoing surveillance project which recruited eligible households from a previous community survey.^4^

Households were recruited between December 2012 and February 2013, for the Mobile Surveillance for Acute Respiratory Infections and Influenza‐Like Illness in the Community (MoSAIC) study, a 5‐year population‐based study of ILI/ARI surveillance. Households in the MoSAIC study were selected using a random sample of participants, previously enrolled in a large community‐based survey (https://www.dbmi.columbia.edu/impact/wicer/). To be eligible for the MoSAIC study, households had three or more members with at least one member under 18 years of age, were Spanish‐ or English‐speaking, and had a cellular telephone with text messaging. During the initial home visit, one volunteer from the household was selected to be the household reporter who received and responded to text messages. The reporter from participating households was sent twice weekly text messages to identify members with symptoms of ARI/ILI. Nasal swabs were collected by research staff in the homes of individuals with ARI/ILI if they fulfilled at least two of the following clinical criteria: fever, runny nose/congestion, sore throat, cough, and/or myalgia. For infants, these criteria were modified to include the sole criterion of runny nose/congestion.^4^ The institutional review board at CUMC (New York, New York) approved the community surveillance study.

### Hospital‐based laboratory surveillance

Hospital samples were collected as part of routine care as medically indicated for adult and pediatric inpatients and outpatients at three CUMC sites (a children's hospital, an acute care hospital serving adults, and a community hospital serving primarily adults) during the same time period as community‐based surveillance. All CUMC sites are located in the same neighborhood as the community surveillance population. At CUMC, testing is recommended only if respiratory symptoms are present and usually is limited to patients requiring hospitalization. The CUMC Institutional Review Board approved this study.

### Laboratory testing

In the community and hospital, respiratory samples were analyzed via multiplex reverse transcriptase polymerase chain reaction (RT‐PCR) using the FDA‐approved FilmArray Respiratory panel 1·7 (Biofire Diagnostics, LLC, Salt Lake City, Utah) that identifies 17 viral and three bacterial respiratory pathogens including adenovirus; coronaviruses (HKU1, NL63, 229E, OC43); human metapneumovirus; rhino/enteroviruses; influenza viruses (A, A/H1, A/H3, A/H1‐2009, B); parainfluenza viruses (1,2,3,4);and respiratory syncytial virus, *Chlamydophila pneumoniae*,* Mycoplasma pneumoniae,* and *Bordetella pertussis* with an overall reported sensitivity of 85‐100% and specificity of 95–100%.^5^ Samples from the community were tested in a research laboratory, and samples from the hospital were tested in the CUMC clinical microbiology laboratory.

### Statistical analysis

We compared respiratory viral activity determined through community‐ versus hospital laboratory‐based surveillance. Overall and monthly viral activities were calculated by dividing total number of positive tests by the total number of respiratory samples tested. Overall positivity rates were compared between the two populations using chi‐squared tests. We also compared distributions of viral positivity, stratified by age between the two groups using chi‐squared tests.

Epidemiologic curves of monthly activity for each virus were created. Statistical concordance between viral activity detected in the community and in the hospital samples was assessed using Pearson's moment correlation coefficients with 95% confidence interval using Fisher transformation. Chi‐squared tests compared: (i) the proportion of individual viral pathogens contributing to viral activity in community vs. hospital surveillance and (ii) the distribution for parainfluenza, coronavirus, and influenza virus types identified in community vs. hospital surveillance.

Finally, to assess bias caused by household clustering of viruses in the community sample, a sensitivity analysis was conducted using only the first case detected in the household for each ARI/ILI episode to determine community viral activity.

## Results

### Study population in community and hospital laboratory surveillance

In the community‐based surveillance study population, 289 households were enrolled between January 2013 and June 2014. The recruited community population included 1407 subjects (mean: 4·9 members/household), with 149 (10·6%) subjects under 5 years of age, 433 (30·7%) subjects aged 5–18 years, 751 (53·4%) subjects aged >18 to 65 years, and 74 (5·2%) subjects aged >65 years. During the study period, 30,219 messages were sent and 22 575 responses obtained (74·7%). There were 955 ILI/ARI episodes reported, for which 865 (90·5%) nasal swabs were obtained (90 participants refused or were missed). Of 865 tested specimens in the community, 551 (63·7%) were positive for ≥1 respiratory pathogen.

Over the same period, 25,350 samples (23 753, 93·7% obtained from inpatients) were tested in the hospital. The 25,350 hospital samples were drawn from 14,988 subjects with 3637 (24·2%) subjects under 5 years of age, 1845 (12·3%) subjects aged 5–18 years, 5554 (37·0%) subjects aged >18 to 65 years, and 3952 (36·4%) subjects aged >65 years. Overall, 10,223 (40·3%) of 25,350 samples in the hospital group were positive for ≥1 respiratory pathogen, a significantly lower proportion of positive results than obtained in community‐based surveillance (*P* < 0·01). Overall monthly positivity ranged from 46·2% to 75·8% (median 63·7%) for community‐based surveillance and from 35·8% to 65·1% (median 53·6%) for hospital‐based surveillance.

### Activity of specific viruses in community‐ and hospital‐based surveillance

Rhinovirus/enteroviruses were the most frequently identified viruses. Ten percent of samples were positive for influenza in the hospital‐ versus 11·3% in community‐based surveillance (Table 1a). Among all positive tests for viral pathogens, 24·9% were positive for influenza in the hospital‐ vs. 17·0% in community‐based surveillance (*P* < 0·01). (Table 1b) The distribution of coronavirus and rhinovirus differed significantly in the two populations, and both were more frequent in the community. In age‐stratified analyses, rhino/enteroviruses were most commonly identified among positive tests, in all age groups except >65‐year‐olds in the hospital group where influenza was more common. Influenza was detected proportionately more in the hospital as compared to the community in all age groups (Table 2).

**Table 1:** (a) Number and proportion of respiratory viruses detected among all tested samples from community‐ and hospital‐based surveillance for acute respiratory illness/influenza‐like illness (b) Number and proportion of respiratory viruses detected among positive tests for viral pathogens from community‐ and hospital‐based surveillance for acute respiratory illness/influenza‐like illness

*Virus*	Community *N* = 865 *n* (%)	Hospital laboratory *N* = 25 350 *n* (%)	*P* value
(a)			
Adenovirus	7 (0·8)	326 (1·3)	0·4
Coronaviruses	95 (10·9)	1134 (4·5)	<0·01
Human metapneumovirus	21 (2·4)	720 (2·8)	0·5
Influenza viruses	98 (11·3)	2540 (10·0)	0·2
Parainfluenza viruses	37 (4·3)	781 (3·1)	0·05
Respiratory syncytial virus	36 (4·2)	1262 (5·0)	0·3
*Rhinovirus/enteroviruses*	281 (32·5)	3402 (13·4)	<0·01

Virus	Community *N* = 575 (%)	Hospital laboratory *N* = 10 165(%)	*P* value
(b)			
Adenovirus	7 (1·2)	326 (3·3)	0·02
Coronaviruses	95 (16·5)	1134 (11·2)	<0·01
Human metapneumovirus	21 (3·7)	720 (7·1)	<0·01
Influenza viruses	98 (17·0)	2540 (24·9)	<0·01
Parainfluenza viruses	37 (6·4)	781 (7·7)	0·3
Respiratory syncytial virus	36 (6·3)	1262 (12·4)	<0·01
Rhinovirus/enteroviruses	281 (48·9)	3402 (33·5)	<0·01

**Table 2:** Number and proportion of respiratory viruses detected among positive tests for viral pathogens by age from community‐ and hospital‐based surveillance for acute respiratory illness/influenza‐like illness

Age	Adenovirus *N* (%)	Coronaviruses	HMPV	Influenza Viruses	Parainfluenza Viruses	RSV	Rhino/enteroviruses
<5 years old							
Community^a^	4 (2·2)	20 (10·9)	12 (6·5)	12 (6·5)	18 (9·8)	16 (8·7)	102 (55·4)
Hospital	222 (5·8)	341 (8·9)	323 (8·4)	469 (12·3)	343 (9·0)	784 (20·5)	1346 (35·2)
5–18 years old							
Community^a^	2 (1·1)	18 (10·3)	3 (1·7)	42 (24·1)	8 (4·6)	8 (4·6)	98 (56·3)
Hospital	47 (3·9)	88 (7·4)	72 (6·0)	351 (29·5)	64 (5·4)	56 (4·7)	512 (43)
>18–65 years							
Community^a^	1 (0·5)	49 (25·3)	6 (3·1)	43 (22·2)	10 (5·2)	10 (5·2)	75 (38·7)
Hospital	23 (0·7)	548 (16·5)	174 (5·2)	1197 (36·1)	152 (4·6)	244 (7·4)	979 (29·5)
>65 years							
Community^a^	0 (0)	4 (22·2)	0 (0)	1 (5·6)	1 (5·6)	2 (11·1)	10 (55·6)
Hospital	5 (0·3)	194 (10·6)	156 (8·5)	706 (38·6)	122 (6·7)	172 (9·4)	475 (26)

a *P* < 0·01 (comparing distributions between community and hospital surveillance).

### Comparison of community‐ and hospital‐based respiratory virus activity 2

**Figure 1:**
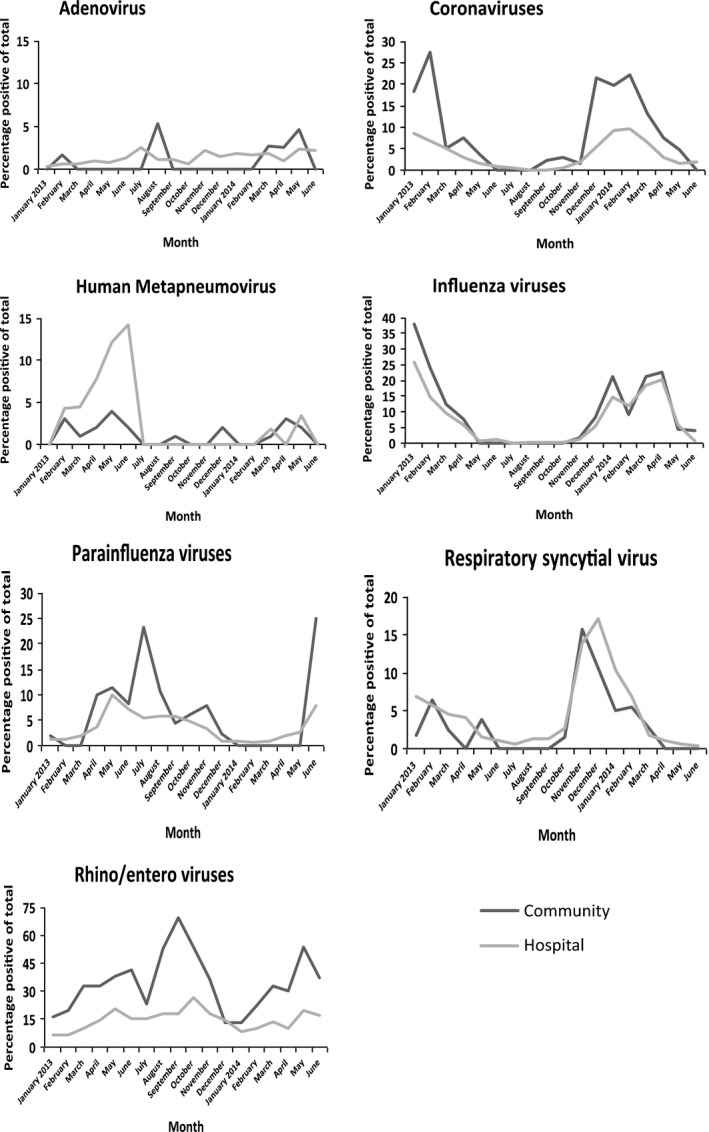
Comparison of Community‐ and Hospital‐based Respiratory Viral Activity from January 2013–June 2014.

The correlation between community‐ and hospital‐based respiratory virus detection by month was strongest for influenza virus and RSV (correlation coefficients of 0·97 and 0·87, respectively) and lowest for adenovirus (0·14) (Table 3 **,** Figure 1).

**Table 3:** Relationship between community‐based and hospital‐based respiratory viral detection by month, January 2013–June 2014

Respiratory Virus	Correlation coefficient (95% CI)
Adenovirus	0·14 (−0·36–0·56)
Coronaviruses	0·88 (0·71–0·96)^a^
Human metapneumovirus	0·64 (0·22–0·84)^a^
Influenza viruses (all subtypes)	0·97 (0·90–0·98)^a^
Parainfluenza viruses (all types)	0·74 (0·29–0·87)^a^
Respiratory syncytial virus	0·87 (0·75–0·87)^a^
Rhinovirus/enteroviruses	0·74 (0·38–0·89)^a^

a *P* value < 0·05.

### Distribution of viral types

Among persons with an influenza virus, influenza B viruses were more common in community than hospital‐based surveillance (51·0% versus 39·7%, *P* = 0·04), while influenza A (H3N2) was more common in hospital than community‐based surveillance (35·4% versus 27·5%, *P* = 0·1). The distributions of parainfluenza virus types and coronavirus types were similar; NL63 was the most commonly isolated coronavirus in both the community and hospital (33·7% and 33·3%, respectively) and type 3 was the most common parainfluenza virus in both groups (48·6% and 55·6%, respectively).

### Sensitivity analysis

When only the first case detected in each household during an ARI/ILI episode was included, the number of positive samples in the community decreased (503/865, 58·1%), but remained significantly higher than in the hospital‐based sample. The relative proportions of viral types detected in the community and hospital and the correlation between community and hospital viral activity remained unchanged (data not shown).

## Discussion

In this study, we demonstrated a significant correlation between community‐ and hospital laboratory‐based surveillance for most respiratory viruses. While previous studies have compared the impact of specific viral etiologies in a hospitalized population versus those seeking care in outpatient clinics, such studies did not fully reflect what might be occurring in the broader community as only medically attended illness was analyzed.^6^, ^7^ Most community testing in the current study reflected non‐medically attended ARI/ILI, as <25% of ill participants sought medical care^4^ confirming the potential of developing mobile health technologies to expand surveillance and outbreak response into the community.

We observed significant differences in the distribution of viruses in the community compared with the hospital. Rhino/enteroviruses and coronaviruses were more frequently detected in community‐based surveillance. We also observed differences in the proportion of influenza strains contributing to community versus hospital activity. The larger proportion of influenza A seen in those hospitalized corroborates existing evidence for increased clinical severity from these strains. Although typically described as having a milder clinical course than influenza A,^8^, ^9^ influenza B is now increasingly described as a cause of mortality and morbidity^10^ and contributed to the preponderance of community activity and a sizeable fraction of hospital activity. The proportions of viruses affecting different age groups in the community versus hospital samples also differed, emphasizing the importance of broad surveillance both by site, community vs. hospital, and by age.

This study has limitations. It was conducted in a select neighborhood in NYC for a limited duration, impacting generalizability. Indications for testing in the hospital‐based analyses were not assessed. Positivity rates were lower in the hospital‐based samples than the community‐based samples, but similar to other hospital studies.^11^ The sensitivity of the multiplex RT‐PCR test could also vary with sample acquisition technique or timing of specimen collection, which may have differed between the two groups. Hospital laboratory testing also included patients who lived outside the neighborhood of the community surveillance cohort, but most reside in NYC, a geographically limited area, which would minimize bias due to variation in neighborhood respiratory viral activity. It is also less likely for patients with ARI/ILI to be referred to our hospital from outside NYC.

In conclusion, we demonstrated a significant association between community‐based surveillance for respiratory viruses using text messaging and hospital laboratory‐based surveillance, although there were some differences in viral distribution between the two settings. Further work will develop detailed temporal associations over a longer time period and explore use of community surveillance to predict hospital resource utilization for ARI/ILI.
